# Case report: First diagnosis of Fabry disease in North Macedonia in a patient presenting with kidney failure on hemodialysis

**DOI:** 10.3389/fgene.2024.1415906

**Published:** 2024-08-14

**Authors:** Nikola Gjorgjievski, Vlatko Karanfilovski, Todor Arsov, Pavlina Dzekova Vidimliski, Galisna Severeova Andreevska, Gjulshen Selim, Petar Dejanov, Vasilena Jordanova, Ivelina Marinova, Emil Paskalev, Igor G. Nikolov

**Affiliations:** ^1^ Faculty of Medicine Ss. Cyril and Methodius in Skopje, University Hospital of Nephrology, Skopje, North Macedonia; ^2^ Faculty of Medical Sciences, University Goce Delcev in Shtip, Stip, North Macedonia; ^3^ Department of Nephrology and Transplantation University Hospital “Alexandrovska”, Sofia, Bulgaria

**Keywords:** Fabry disease, chronic kidney disease, hemodialysis, α-galactosidase A, X-linked disorder

## Abstract

**Introduction:**

Fabry disease is a rare X-linked lysosomal storage disorder caused by α-galactosidase A (α-Gal A) deficiency. Reduced or absent enzyme activity causes progressive lysosomal accumulation of globotriaosylceramide (Lyso-Gb3) in various cells throughout the body to trigger inflammation and fibrosis.

**Case description:**

We present the first familial case of Fabry Disease in North Macedonia identified based on clinical manifestations and confirmed through enzyme, biomarker, and genetic tests. The index case in the family was a 45-year-old male undergoing hemodialysis therapy. He has had chronic burning uncontrolled limb pain since childhood, intermittent abdominal cramps, anhidrosis, and hypertension. The constellation of clinical presentations accompanied by similar symptoms in close family members prompted the enzyme, biomarker, and genetic analyses for Fabry disease. Genetic testing identified a known pathogenic *GLA* missense variant c.443G>A or p.(Ser148Asn) in the hemizygous state. Subsequent family studies allowed identification of another hemizygous male and five heterozygous female carriers affected by this X-linked disorder.

**Conclusion:**

We report identification of the first familial case of Fabry disease in North Macedonia and describe the phenotype associated with the Ser148Asn *GLA* variant. Greater awareness of this rare disease linked to continuous medical education is crucial for timely diagnosis and treatment.

## Introduction

Fabry–Anderson or Fabry disease (FD, OMIM#301500) is a rare X-linked lysosomal storage disorder resulting from the absence or impaired function of α-galactosidase A (GLA, OMIM*300644), an enzyme responsible for the catabolism of glycosphingolipids by hydrolysis of the terminal α-galactosyl moieties from glycolipids and glycoproteins. This leads to progressive lysosomal accumulation of globotriaosylceramide (Lyso-Gb3) in the cells throughout the human body and subsequent multiorgan failure (Michaud et al., 2020). This progressive and lifelong metabolic condition affects both genders (Michaud et al., 2020) and was first reported in 1897 before being later classified as sphingolipidosis (Anderson, 1898; Fabry, 1898). The biosynthesis of GLA is under the control of the *GLA* gene, which is located on chromosome Xq22.1 (Anderson, 1898); it contains seven exons with 1,290 coding nucleotides that encode a 49 kDa protein consisting of 429 amino acid residues, including a signal peptide of 31 residues (Duro et al., 2018).

The Human Gene Mutation Database and ClinVar database (Stenson et al., 2020; ClinVar, 2024) report about 500 pathogenic *GLA* variants, including point missense and non-sense substitutions, duplications, deletions, splice sites, and complex mutations (Desnick, 2020). Genotype–phenotype correlations are generally poorly understood in FD because most patients have private pathogenic genetic variants. More extensive clinical studies with unrelated patients having the same genotype are required to gain further understanding of these relationships. It is well-known that complete loss of enzyme function leads to the *classical early-onset form* and reduced enzyme activity is associated with the *late-onset form* of FD; most patients with cardiac and kidney presentations also have missense variants with residual enzyme activities (Valtola et al., 2020; Chimenz et al., 2022). FD is a multisystem disorder with a wide spectrum of clinical symptoms due to mitochondrial dysfunction, blood vessel wall thickening, and endothelial dysfunction with microvessel luminal occlusions or thrombosis, which lead to progressive cardiovascular, cerebrovascular, and kidney dysfunctions (Paim-Marques et al., 2022).

There are two major forms of FD, which are the classical and late-onset variants. The classical form is usually seen in hemizygous males with enzyme activities below 1% and very rarely in heterozygous females. The non-classical or late-onset form is characteristic of males with enzyme activities exceeding 1% and is the most common type manifesting in females, which is related to skewed X-chromosome inactivation (Stepien et al., 2023).

The characteristic symptoms and signs of the *classical form* among children and adults are peripheral neuropathy with chronic burning as well as uncontrolled and unrelenting pain in the limbs (acroparesthesis) accompanied by abdominal cramps (Chimenz et al., 2022). Moreover, hypohidrosis or anhidrosis with dry skin, hot and cold exercise intolerance, angiokeratoma, and cornea verticillata are observed during childhood (Chimenz et al., 2022). Cardiovascular disorders (arrhythmias, myocardial infarction, heart failure, and sudden death), kidney disorders (microalbuminuria, proteinuria, glomerular hyperfiltration, and kidney failure), and central and autonomic nervous systems damage associated with the dorsal root ganglia (early stroke and transitory ischemic attacks) are the most common causes of morbidity and mortality in patients with FD. The late-onset form may not present with any symptoms until a later age, but it may still progress to life-threatening complications (Stepien et al., 2023).

Recognizing the constellation of clinical symptoms and signs will lead to clinical suspicion, which can be assessed by confirmatory enzyme, biomarker, and genetic tests (Wanner et al., 2023); however, the rarity of the condition and late-onset forms with atypical presentations can make diagnosis very challenging and may often require lengthy diagnostic processes. Introducing guidelines for appropriate screening in high-risk populations would therefore be helpful for identifying patients and their relatives at early or presymptomatic stages of the disease (Wanner et al., 2023). In this article, we describe the first diagnosed case of FD in North Macedonia, including six additional symptomatic and presymptomatic relatives in this family who carry the identified pathogenic *GLA* variant.

## Case description

A 45-year-old male was admitted to the University Hospital of Nephrology, Skopje, North Macedonia, with kidney failure (KF) requiring hemodialysis (HD). The patient presented with dysuria, anemia (Hgb 78 g/L, reference value: 120–180 g/L), proteinuria (2.2 g/24 h, reference value: < 0.2 g/24 h), high level of blood nitrogen urea (BUN 45 mmol/L, reference value: 2.7–7.8 mmol/L), elevated serum creatinine (1,193 μmol/L, reference value: 45–109 μmol/L), hyperkalemia (5.9 mmol/L, reference value: 3.8–5.5 mmol/L), hyperphosphatemia (2.14 mmol/L, reference value: 0.8–1.4 mmol/L), hypocalcemia (1.9 mmol/L, reference value: 2.1–2.6 mmol/L), and muscle pains. The patient began chronic HD therapy via a central femoral vein catheter and received transfusions with filtered red blood cells. He has had a 10-year medical history of high blood pressure that was treated with antihypertensive drugs and gradual increases in BUN and serum creatinine levels with subnephrotic range of proteinuria over the last 2 years; however, he had not consulted a nephrologist. From an early age, he presented with uncontrolled burning chronic pain in the limbs, intermittent abdominal pain, as well as cold and heat intolerance.

An ultrasound examination showed small-sized kidneys (right kidney 86 × 42 mm and left kidney 86 × 43 mm) with hyperechogenic parenchyma (12–13 mm), and the electrocardiogram showed sinus tachycardia (heart rate: 110/min) with inverse T wave in the precordial leads. The patient was discharged from the hospital with an initial diagnosis of KF requiring regular HD. Over the next 6 months, the patient underwent further examinations to prepare him for live-donor kidney transplantation from his sister. During this period, nephrologists who had recently completed a training course on FD in a specialized center in Bulgaria suggested additional testing for FD based on the early onset of KF and history of acroparesthesias, abdominal cramps, anhidrosis, and cornea verticillata. Cardiac magnetic resonance imaging (MRI) demonstrated concentric hypertrophic cardiomyopathy with end-diastolic thickness of the interventricular septum and posterior wall. A more detailed examination of the family history revealed similar symptoms in his daughter (acroparesthesias, abdominal cramps, and anhidrosis) and in a maternal cousin who was also a kidney transplant recipient with a history of acroparesthesias, abdominal cramps, and anhidrosis. The enzyme and biomarker tests showed almost undetectable GLA activity (0.1 μmol/L/h, normal: >2.8 μmol/L/h) and greatly elevated Lyso-Gb3 (73.3 ng/mL, normal: <3.5 ng/mL). The genetic testing identified a previously reported pathogenic *GLA* hemizygous variant NM_000169.3 (GLA): c.443G>A or p.(Ser148Asn) (Ashton-Prolla et al., 2000), ClinVar ID 496827, which was absent in the population databases. The enzyme, biomarker, and diagnostic genetic tests of the proband and subsequent cascade testing in the family were performed using dried blood spots in ARHIMEDlife^®^, a commercial diagnostic laboratory accredited according to the ISO 15189 standard. Subsequently, the patient underwent a successful kidney transplantation from his non-carrier sister.

## Familial study

**FIGURE 1:**
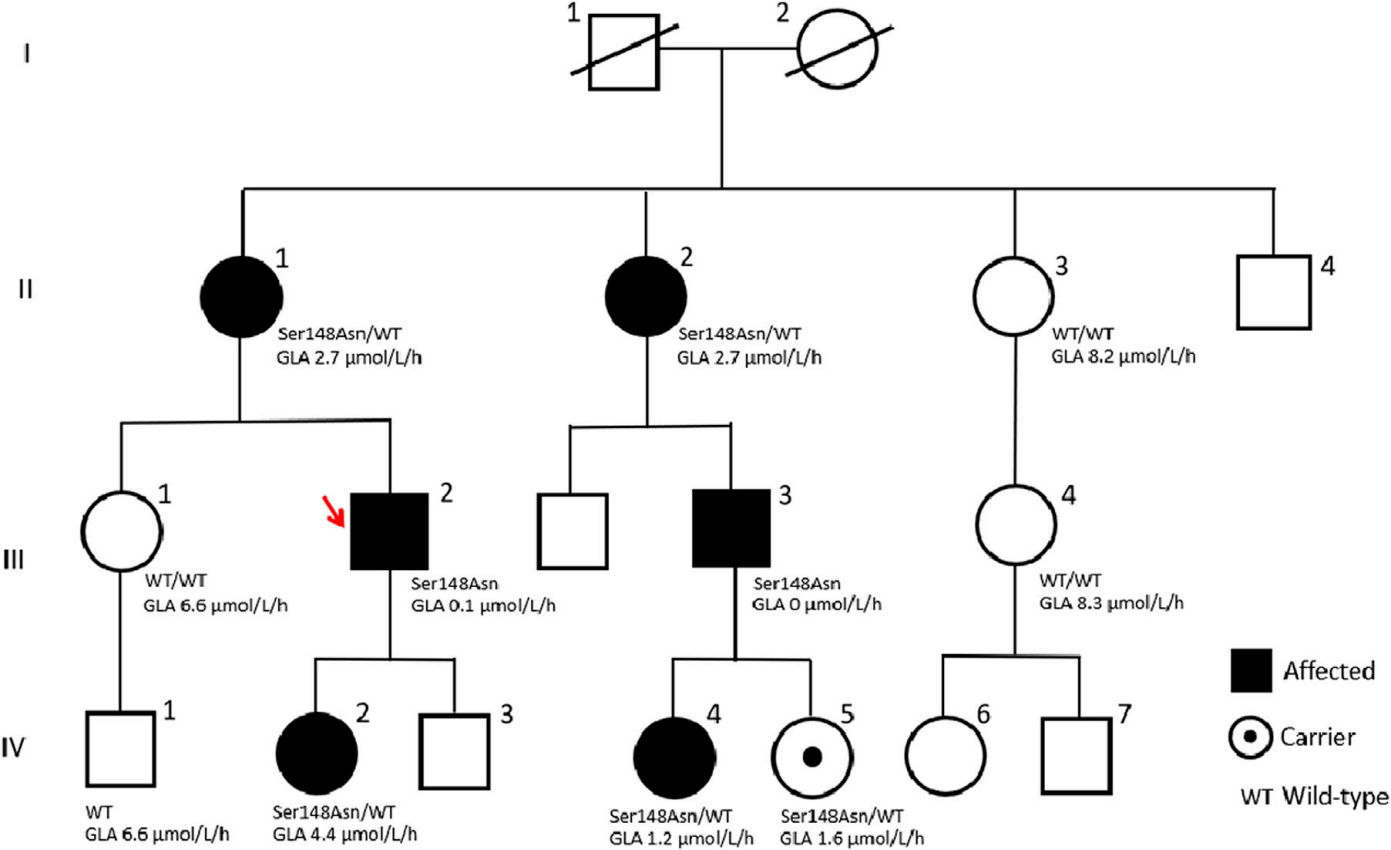
Pedigree tree of the first Macedonian family identified with Fabry disease.

A detailed family history identified 18 individuals across three generations in the patient’s pedigree (Figure 1). Personal histories, clinical findings, and subsequent enzyme, biomarker, and genetic analyses (Table 1) identified another six affected cases (positive for the *GLA* variant). The proband’s 14-year-old daughter (IV-2) presented specific clinical manifestations from an early age, including cornea verticillata, acroparesthesia, abdominal cramps, and anhidrosis. Her laboratory examinations demonstrated microalbuminuria (30 g/L), intermittent proteinuria, normal GLA activity, and mildly elevated level of Lyso-Gb3 (Table 1).

**TABLE 1:** Presentation of the enzyme activities, Lyso-Gb3 levels, and clinical manifestations of all members of the family.

Individual	Age/Gender	Genotype	α-galactosidase activity (reference: >2.8 μmol/L/h)	Lyso-Gb3 (reference: <3.5 ng/mL)	Clinical manifestation	Relationship to proband
III-2	45/M	p.Ser148Asn hemizygote	0.1 μmol/L/h	73.3. ng/mL	Dialysis Acroparesthesias Anhidrosis Cardiomyopathy	
III-3	44/M	p.Ser148Asn hemizygote	0.0 μmol/L/h	88.4 ng/mL	Dialysis Acroparesthesias Anhidrosis Cardiomyopathy	Maternal cousin
IV-2	14/F	p. Ser148Asn heterozygote	4.4 μmol/L/h	5.3. ng/mL	Acroparesthesias Abdominal cramp Cornea verticillata Intermittent proteinuria Microalbuminuria	Daughter
II-1	75/F	p. Ser148Asn heterozygote	2.7 μmol/L/h	7.3 ng/mL	Cardiomyopathy CVI	Mother
II-2	70/F	p. Ser148Asn heterozygote	2.7 μmol/L/h	5.4 ng/mL	Cardiomyopathy CVI	Maternal aunt
IV-4	18/F	p. Ser148Asn heterozygote	1.2 μmol/L/h	8.3 ng/mL	Microalbuminuria	Niece of maternal cousin
IV-5	9/F	p. Ser148Asn heterozygote	1.6 μmol/L/h	5.9 ng/mL	Asymptomatic	Niece of Maternal cousin
II-3	81/F	wild type	8.2 μmol/L/h	3.5 ng/mL	Cardiomyopathy CVI	Maternal aunt
III-4	60/F	wild type	8.3 μmol/L/h	1.7 ng/mL	Hypertension	Maternal cousin
III-1	52/F	wild type	6.6 μmol/L/h	2.1 ng/mL	Hypertension	Sister
IV-1	32/M	wild type	5.5 μmol/L/h	1.8 ng/mL	Asymptomatic	Nephew

The proband’s cousin (III-3) received a kidney transplant 5 years before based on a finding of focal segmental glomerulosclerosis (FSGS) or IgA nephropathy from a kidney biopsy many years prior to KF; he also showed acroparesthesias, anhidrosis, and sinus tachycardia. His cardiac MRI demonstrated concentric hypertrophic cardiomyopathy with end-diastolic thickness of the interventricular septum and posterior wall, which is a typical finding in FD. He had undetectable GLA activity and greatly elevated Lyso-Gb3 (Table 1). The four other carrier females in this family had borderline (II-1 and II-2) or reduced levels (IV-4 and IV-5) of enzyme activities and slightly elevated Lyso-Gb3 levels (Table 1). The remaining four members of the family proved to be non-carries of the pathogenic *GLA* variant, with normal enzyme activities and normal Lyso-Gb3 levels.

## Discussion

Rare diseases affect more than 400 million people globally, and their timely diagnosis and treatment remain challenging, especially in countries with limited health resources (Verma et al., 2022). The affected individuals in under-resourced countries usually experience lengthy diagnostic odysseys, during which life-threatening complications may occur sometimes (Verma et al., 2022). On average, patients with FD are examined by 7–10 different specialists before an accurate diagnosis is made (Kudumija et al., 2007). Studies have shown that the diagnostic procedures can be very lengthy: 13 years for men and 17 years for women (Hoffmann and Mayatepek, 2009). The reasons for this are the rarity of the condition, heterogeneous and variable clinical course of FD, especially in women, and broad range of possible differential diagnoses (Hoffmann and Mayatepek, 2009). Newborn screening for FD is important because children who are diagnosed and treated early may have normal growth and development. However, healthcare systems in the United States, United Kingdom, Canada, several countries in Europe, Taiwan, and Japan have well-developed and implemented newborn screening programs (Gragnaniello et al., 2023). Thus far, FD is not part of the national newborn screening program in North Macedonia.

In this geographical region, the first cases of FD were identified much earlier than the current family, for example, in Bulgaria in 1963, in Slovenia in 1991, and in Serbia in 2009 (Kotnik et al., 2005; Paskalev et al., 2011; Ćelić et al., 2022). Previously, efforts to individually test for FD were performed in highly suspect patients in North Macedonia many years ago. One of these patients had a positive test, but further investigation and family screening for the disease were not pursued, and the case was not reported as well. High awareness and proper education are essential for early recognition, diagnosis, understanding, and management of FD. Therefore, close cooperation with centers with higher expertise in FD is crucial for early diagnoses and treatments of patients with FD in developing countries.

In our case, the index patient was identified through enzyme, biomarker, and genetic tests after preliminary findings of KF and cardiomyopathy, which were clinically highly suspect for FD, and the remaining cases were confirmed through family screening and pedigree analysis. The first cases of FD were similarly identified in Serbia and Bulgaria, where later screening among the high-risk patients on HD was also carried out (Paskalev et al., 2011; Ćelić et al., 2022). The prevalence of FD is estimated to be 1 in 17,000–117,000 newborns (Gragnaniello et al., 2023), and according to the population of North Macedonia (census 2021: 1,836,713 inhabitants), the estimated number of patients with FD would be 16 to 46 individuals; here, the lower end of the range would possibly indicate the number of patients with the classical form and the higher end of the spectrum could include the milder forms of FD.

We identified a Macedonian family with seven affected family members who carried the pathogenic *GLA* variant Ser148Asn. Both males in the family had the classical form of FD, with less than 1% enzyme activity and markedly elevated levels of Lyso-Gb3. They both developed end-stage kidney disease, requiring kidney replacement therapy in the fourth decade of their life. The same *GLA* genetic variant had previously been described in patients with the classical FD phenotype, including male individuals with cardiomyopathy and KF requiring dialysis as well as females with less-severe symptoms and longer life expectancies (Ashton-Prolla et al., 2000). The *GLA* variant identified in the present family has been previously reported in two brothers with classical FD and shown in the transfection study to reduce GLA enzyme activity to under 0.1% compared to the controls (Shimotori et al., 2008). Another study identified this *GLA* variant in a 43-year-old male patient with classical FD having serum creatinine level of 3 mg/dL (265 μmol/L), renal cortical cysts, increased renal echogenicity, and decreased corticomedullary differentiation (Ronald et al., 2004). Two studies reported two male patients with classical FD hemizygous for different missense substitutions affecting the same amino acid residue Ser148Arg; one of these patients had reduced GLA levels of 0.4 U/mL, while the other was a 37-year-old patient having a creatinine level of 4 mg/dL (353 μmol/L) with ultrasound/MRI findings of renal cortical cysts, parapelvic cysts, increased renal echogenicity, scarring, and decreased corticomedullary differentiation. Interestingly, the same study also reported a 36-year-old female with the same *GLA* variant (presumably a female relative) with borderline serum creatinine level of 1.2 mg/dL (106.1 μmol/L) and MRI findings of renal cortical cysts, renal atrophy, and decreased corticomedullary differentiation (Topaloglu et al., 1999).

Female FD patients can present with a wide range of clinical symptoms, and the severity of the manifestation depends on the degree to which the normal X-chromosome is inactivated in various tissues (MacDermot et al., 2001). It was found that up to 90% of heterozygous females may have mild clinical manifestations of FD, while up to 30% exhibited multiple and serious disease manifestations, including transient ischemic attacks, stroke, and KF (MacDermot et al., 2001).

Our study describes the clinical features of five female patients heterozygous for the Ser148Asn genetic variant. The most severe clinical presentation was in a 14-year-old girl, who presented with acroparesthesias, abdominal cramps, cornea verticillata, and microalbuminuria despite having normal enzyme activity. The symptoms began from an early age as inexplicable abdominal pains, burning chronic pain in the limbs with transient edema. This finding corresponded with data from the Fabry Outcome Survey on 358 female patients, which showed that enzyme activities in the peripheral blood leukocytes did not correlate with disease severity in females (Mehta et al., 2004). Two female patients had cardiomyopathy and cerebrovascular incidents later on in life (above 70 years), one had isolated microalbuminuria (30 g/L) at the age of 18, and only one out of the five female patients in our series was asymptomatic at 9 years of age.

Therapy for FD includes enzyme replacement and non-enzyme-based medications (Besekar et al., 2023). Enzyme replacement therapy (ERT) with recombinant human GLA enzyme (Agalsidase-α or Agalsidase-β) improves the clinical signs and symptoms of FD, such as kidney dysfunction, neuropathic pain, gastrointestinal disorders, and cardiac manifestations; the treatment reduces Lyso-Gb3 accumulation and delays organ damage (Biegstraaten et al., 2015). In 2015, the European Fabry Working Group published the recommendations for initiation and cessation of ERT in patients with FD (Biegstraaten et al., 2015); in 2020, the opinion-based PREDICT-FD modified Delphi consensus initiative considered the early indicators of disease progression in FD for disease-specific treatment initiation (Hughes et al., 2020). According to these recommendations and consensus initiatives, initiation of therapy is advised in all males with classical FD as soon as there are early clinical indicators of kidney, heart, or brain involvement, but should be considered in classical male patients ≥16 years of age even in the absence of clinical signs or symptoms of organ involvement. In classically affected females and males with non-classical FD, treatments should begin as soon as there is early clinical evidence of kidney, heart, or brain involvement, but may be considered in females with non-classical FD with early clinical signs that are considered to be due to FD (Biegstraaten et al., 2015; Hughes et al., 2020). However, there is a general agreement in literature indicating the benefits of early treatment in FD. As an alternative to intravenous ERT, the administration of migalastat, a first-in-class oral chaperone therapy, has shown favorable outcomes in FD patients with amenable mutations (Gjorgjievski et al., 2023). The Ser148Asn variant detected in our patients is not amenable to migalastat (McCafferty and Scott, 2019), and The National Commission for Rare Diseases approved the initiation of ERT for the two male patients (but not for the symptomatic female patient), according to the criteria proposed by the European Fabry Working Group. However, our aim is to provide comprehensive therapy for every affected member based on medical evidence.

In conclusion, the diagnosis of FD is a great challenge for clinicians and requires a high degree of knowledge of the disease as well as detailed family histories of highly suspicious cases (Wanner et al., 2023). This report provides a detailed description of the clinical symptoms, signs, age of onset, and evolution over time in multiple family members carrying the same pathogenic mutation associated with FD, which is a small contribution to the establishment of genotype–phenotype correlation (Table 2). Enzyme, biomarker, and genetic tests of dried blood spot samples facilitate wider applications of such testing procedures. In the future, it will be necessary to conduct a more comprehensive screening of the clinically suspected patients in our country, including patients with KF on kidney replacement therapy. We believe that the formation of a multispecialty team would contribute to the early diagnosis and timely initiation of therapies in patients with FD to prevent permanent organ damage.

**TABLE 2:** Clinical manifestation of all members of the family based on affected organs.

	Age and gender	Kidney	Heart	Skin	GIT	CNS and nerve	Eye and ear
III-2	45/M	+	+	-	+	+	-
III-3	44/M	+	+	-	+	+	-
IV-2	14F	+	-	-	+	+	+
II-1	75/F	+	+	-	-	-	-
II-2	70/F	+	+	-	-	-	-
IV-4	18/F	+	-	-	-	-	+
IV-5	9/F	-	-	-	-	-	-

^a^ GIT, gastrointestinal system; CNS, central nervous system.

## Data Availability

The original contributions presented in the study are included in the article/supplementary material, further inquiries can be directed to the corresponding author.

## Abbreviations

FD, Fabry disease; GLA, α-galactosidase A; Lyso-Gb3, globotriaosylceramide; KF, kidney failure; HD, hemodialysis; FSGS, focal segmental glomerulosclerosis; Ser, serine; Asn, asparagine; ERT, enzyme replacement therapy.
